# CodaChrome: a tool for the visualization of proteome conservation across all fully sequenced bacterial genomes

**DOI:** 10.1186/1471-2164-15-65

**Published:** 2014-01-24

**Authors:** Joe Rokicki, David Knox, Robin D Dowell, Shelley D Copley

**Affiliations:** 1 Department of Molecular, Cellular and Developmental Biology, University of Colorado at Boulder, Boulder CO, USA; 2 BioFrontiers Institute, University of Colorado at Boulder, Boulder CO, USA; 3 Computational Biosciences Program, University of Colorado Anschutz Medical Campus, Aurora CO, USA

## Abstract

**Background:**

The relationships between bacterial genomes are complicated by rampant horizontal gene transfer, varied selection pressures, acquisition of new genes, loss of genes, and divergence of genes, even in closely related lineages. As more and more bacterial genomes are sequenced, organizing and interpreting the incredible amount of relational information that connects them becomes increasingly difficult.

**Results:**

We have developed CodaChrome (http://www.sourceforge.com/p/codachrome), a one-versus-all proteome comparison tool that allows the user to visually investigate the relationship between a bacterial proteome of interest and the proteomes encoded by every other bacterial genome recorded in GenBank in a massive interactive heat map. This tool has allowed us to rapidly identify the most highly conserved proteins encoded in the bacterial pan-genome, fast-clock genes useful for subtyping of bacterial species, the evolutionary history of an indel in the *Sphingobium* lineage, and an example of horizontal gene transfer from a member of the genus *Enterococcus* to a recent ancestor of *Helicobacter pylori*.

**Conclusion:**

CodaChrome is a user-friendly and powerful tool for simultaneously visualizing relationships between thousands of proteomes.

## Background

All recognized living organisms are related by virtue of descent from the last universal common ancestor (the LUCA). This connection is often visualized with phylogenetic trees constructed from alignments of 16S rRNA sequences [1,2]. Such trees are useful for establishing patterns of vertical descent and for genus- and species-level sorting of organisms present within communities. However, they are less useful for exploring relationships between organisms at the level of individual genes. Gene loss, duplication and divergence of genes, horizontal gene transfer, movement of insertion sequences, and the continuous onslaught of bacteriophages remodel bacterial genomes extensively. The variability in genomes with nearly identical 16S ribosomal sequences was a considerable surprise when, for example, the genome of *E. coli* O157 was sequenced and compared to that of *E. coli* K12 [3], but has become recognized as a common phenomenon as multiple genomes from genera such as *Escherichia*[4], *Salmonella*[5], *Streptococcus*[6], *Yersinia*[7] and *Sphingobium*[8] have been compared.

Thousands of bacterial genomes are currently recorded in GenBank, providing a rich source of information about evolutionary processes that shape genomes, adaptation to specific environmental niches, the composition of the core genomes and pan-genomes of microbial species, and the emergence of novel proteins by both divergent and convergent evolution. Mauve has been widely used to analyze rearrangements and horizontal gene transfers that have occurred as microbial genomes have evolved [9], but visualization of relationships becomes difficult for more than a small number of genomes because hundreds of translocations and inversions are often seen even between genomes of closely related species [10]. Mugsy can be used to align larger numbers of genomes, but its application is limited to closely related genomes [11]. However, there are currently no tools for simultaneous analysis and visualization of the relationships between the genomes of thousands of distantly related organisms. CodaChrome was designed to fill this critical gap. CodaChrome assesses relationships between proteins rather than genes because amino acid identities provide a more robust signal at high levels of divergence. These proteomic relationships are visualized as a massive interactive heat map in which the proteins are ordered according to the order of genes within a seed genome. This organization allows for straightforward comparisons of thousands of species regardless of the inversions or translocations that would complicate a genomic sequence alignment.

Here we describe how CodaChrome works and give several examples of how it can be used to address questions of biological interest. For example, patterns that emerge in CodaChrome heat maps indicate proteins that are particularly highly conserved for functional reasons, as well as proteins that are rapidly diverging and useful for subtyping of bacterial species. Other unique patterns that are generated by horizontal gene transfers or gene loss within certain lineages provide new insights into the evolutionary history of bacterial proteins. This method of visualization could easily be extrapolated to other domains of life.

## Implementation

### Generation of the CodaChrome matrix file

CodaChrome consists of a series of PERL scripts that generate a CodaChrome matrix file and a user-friendly graphical user interface (GUI) that renders the CodaChrome matrix file into an interactive heat map. To generate the matrix file, the PERL scripts retrieve the protein sequences encoded by every fully sequenced bacterial genome recorded in GenBank and construct a BLAST database. Several pre-computed matrix files generated using the 2708 complete bacterial genomes as of 12/3/2013 are available at http://www.sourceforge.com/p/codachrome. The PERL scripts can be used to generate updated or custom matrix files. When the user selects a “seed organism”, each protein encoded in the seed organism is individually queried against the previously generated BLAST database. All statistically significant alignments (E-value < 1e-20) are recorded in a massive list containing hundreds of alignments for each of the thousands of proteins in a typical bacterial proteome. The list of significant alignments is then consolidated and reorganized into a labeled and tab-delimited matrix of best matches. We note that by taking the best BLAST hit we are intentionally targeting the closest homolog based on sequence identity rather than necessarily attempting to identify the closest ortholog [12]. Each column of the matrix corresponds to a protein in the seed proteome, ordered as its corresponding gene is ordered in the seed genome. Each row corresponds to the set of proteins encoded by a specific chromosome or plasmid represented in the BLAST database. The matrix is populated with the percent identities of pairwise alignments between the seed protein, indicated by the column, and the “best hit” encoded by the plasmid or chromosome indicated by the row. The resulting matrix file is a concise summary of the relationship between the seed proteome and the proteomes encoded by all of the fully sequenced bacterial genomes in GenBank.

### Visualization of the CodaChrome matrix file

**Figure 1:**
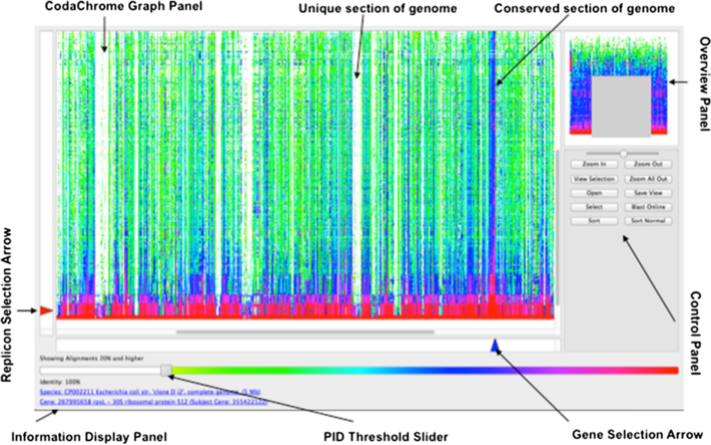
**The CodaChrome GUI.***Salmonella enterica 14028S* was loaded as the seed organism. The rows were sorted by average proteome identity. The portion of the heat map visualized in the CodaChrome Graph Panel is indicated by the grey box in the Overview Panel. Lighter-than-normal vertical stripes represent large clusters of proteins unique to the seed organism. Dark vertical stripes represent clusters of highly conserved proteins. Buttons embedded in the control panel allow the user to interact with the visualization of the matrix file. A slider at the top of the control panel allows the user to zoom in or out. Replicon arrows and gene selection arrows indicate the alignment selected and described in the information display panel. Finally, the percent identity threshold slider allows users to filter alignments below a specified threshold. For this image, the threshold was set to 20%. The slider also functions as a legend indicating how percent identities are translated into color.

The data contained in the CodaChrome Matrix File can be visualized using the CodaChrome graphical user interface (GUI) (Figure 1). This GUI is programmed in C++/QT and can be compiled to run on most common platforms. It renders the CodaChrome matrix file into a heat map image in which each row corresponds to a replicon in GenBank, each column corresponds to a protein in the seed organism and each pixel is colored according to the percent identity between the two proteins it represents.

The GUI allows users to interact with data represented by this image in many ways. Users may zoom in or out. They may click on the pixels that represent individual alignments to identify which protein-to-protein comparison is represented. They may adjust a threshold to filter out pixels representing low-percentage-identity alignments. They may locate a specific protein or species of interest by typing its name into a dialog box. Finally, they may sort the rows of the matrix by the average percent identity to the seed proteome, to a subset of adjacent seed proteins, or to a single seed protein of interest.

### The CodaChrome scaling algorithm

The number of pairwise alignments recorded in a CodaChrome matrix file often exceeds the number of pixels on a typical computer monitor. To visualize the CodaChrome heat map in its entirety, it must be scaled such that each pixel of the scaled image represents multiple elements of the heat map.

Scaling is accomplished in typical graphics processing algorithms by averaging the values of a block of pixels and coloring a single representative pixel with this value. Alternatively, a single pixel within the block can be chosen at random to represent the whole. Both of these algorithms are unacceptable for CodaChrome because they result in the eclipse of interesting, high-value elements by nearby and more common low-value elements. Instead, CodaChrome implements a custom max sampling algorithm that represents a field of pixels with a single pixel of the maximum value within the field. This algorithm causes high-percentage values to “rise to the top” as a user zooms out. This algorithm allows even a single high-percentage value to be visualized while the image is zoomed out by many factors.

## Results and discussion

### An overview of the CodaChrome heat map

CodaChrome generates a BLAST database [13] of all proteins encoded by every finished bacterial genome recorded in GenBank. This database is queried with each protein encoded by a user-selected “seed genome”. The pairwise identity between each seed protein and the best match in each organism in the database is indicated by color in an interactive heat map (Figure 1). Each column of the heat map corresponds to a protein in the seed organism. The columns are ordered according to the positions of the corresponding genes in the seed genome. Each row of the heat map corresponds to a set of proteins encoded by a chromosome or plasmid in GenBank. The color of each element in the heat map indicates the percent identity shared between the seed protein, indicated by the column, and the most similar protein encoded by the chromosome or plasmid, indicated by the row. When visualized in this manner, the massive amount of information in the hundreds of thousands of BLAST alignments comparing one proteome to all other proteomes becomes an easily navigable tartan of color, in which patterns emerging above the background reveal important biological relationships and evolutionary events. Because the heat map preserves the gene order of the seed organism, conclusions about genomic events such as insertions and deletions may be inferred even though only protein sequences are compared. The rows of the heat map may be ordered by average percent identity to the entire seed proteome, to a subset of adjacent seed proteins, or to a single seed protein of interest. This method of proteome comparison is represented schematically in Additional file 1: Figure S1. Note that the patterns in the CodaChrome heat map will differ depending upon which proteome is used as the seed and upon the ordering of the rows. The effects of different parameter choices can be seen by opening two instances of Codachrome and displaying them side-by-side in adjacent windows on the computer screen.

In developing CodaChrome, we made certain trade-offs to present the most useful information and the most accurate representation of proteomic relationships. First, the CodaChrome BLAST database is composed only of protein sequences encoded in finished bacterial genomes. The advantage of this method is that users can be confident that gaps or absences in expected patterns represent actual absences of proteins from the organism indicated and not gaps in an incomplete genome assembly. Second, rather than lumping all of the replicons of a given genome together into a single row, CodaChrome assigns each plasmid and chromosome within a genome to its own row. Because the replicons are on separate rows, events such as translocations between replicons or the integration of a plasmid into a chromosome are evident. Third, CodaChrome visualizes the percent identities of amino acid alignments rather than nucleic acid alignments. This choice eliminates the possibility of examining relations between intergenic and other non-coding regions of the genome, but allows CodaChrome to visualize relationships over greater evolutionary distances [14]. Finally, CodaChrome only visualizes the relationships of proteins encoded by the seed genome. Proteins in other organisms that are not found in the seed organism will not be represented in the heat map.

Figure 1 illustrates the CodaChrome graphical user interface (GUI) with *Salmonella enterica* 14028S loaded as the seed organism and with the rows ordered by average percent identity to the seed proteome. The protein identity (PID) threshold slider allows the user to adjust a percent identity cutoff to filter information being displayed as well as indicates the color values corresponding to different levels of percent identity: for example, yellow-green corresponds to 20%, blue corresponds to 70% and red corresponds to 100% identity. The predominantly red and pink band at the bottom of the heat map represents the set of proteomes in which many proteins have nearly 100% identity to the proteins of the seed proteome. Above this band is a sea of green and blue corresponding to proteins from bacteria with more distant relationships to the seed organism. This background of blue and green is lined with several vertical stripes that are unusually dark or unusually light. These stripes correspond to sections of the seed proteome that are especially conserved or especially unique, respectively, in the proteomes in the database.

In the following sections, we show how CodaChrome can be used to address a range of biologically interesting questions. In each case, CodaChrome can provide an answer within a few minutes that previously would have taken considerably more time and/or sophisticated programming. We will also show how anomalous patterns in the CodaChrome heat map can reveal previously unrecognized relationships; behind each of these is an untold biological story.

### Identification of the most highly conserved proteins in the bacterial biosphere using CodaChrome

High sequence conservation in proteins from widely divergent organisms is an indication of extreme “purifying” selection; relatively few sequence changes have been tolerated over billions of years. High levels of sequence conservation suggest that the function of the protein is particularly critical for survival, and furthermore that performance of this function constrains its amino acid sequence as a result of specific requirements on correct positioning of residues in sites involved in ligand recognition, enzymatic activity, and/or interactions with other proteins or macromolecules.

CodaChrome provides an excellent way to identify the most highly conserved proteins in bacteria. After sorting by whole proteome percent identity and applying a percent identity threshold, proteins whose sequences are highly conserved appear as pillars of red and blue that are present even in evolutionarily distant organisms with few other highly conserved homologs.

**Figure 2:**
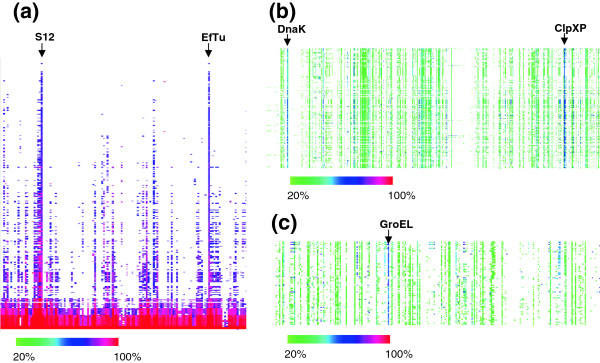
**Identification of extremely highly conserved proteins using CodaChrome. (a)** A segment of the CodaChrome heat map with *Salmonella enterica* subsp. enterica serovar Typhimurium 14028S loaded as the seed sequence and the PID threshold set at 75%. Dark pink and red columns representing pair-wise comparisons of proteins S12 and EF-Tu are indicated. **(b)** Enlargement of a different region of the same CodaChrome heat map as in the previous panel. PID threshold was set to 20%. The dark column on the right represents two adjacent highly conserved proteins ClpX and ClpP. The dark column on the left represents DnaK. **(c)** Enlargement of another region of the same CodaChrome heat map as in panel **a**. PID threshold is set to 20%. The region is centered on the highly conserved protein GroEL.

Two of the most highly conserved proteins in bacteria are elongation factor EF-Tu and the 30S ribosomal subunit protein S12. Figure 2 shows that these two proteins are considerably more conserved than the majority of ribosomal proteins, which themselves are among the most highly conserved proteins and account for most of the other pillars in the heat map. EF-Tu and S12 are both involved in ensuring the fidelity of protein translation [15]. EF-Tu escorts aminoacyl tRNAs to the A-site of the ribosome. If codon-anti-codon pairing is correct, hydrolysis of GTP results in a conformational change that completes the delivery of the charged tRNA into the A-site and causes dissociation of EF-Tu. S12 lies near the A-site at the interface between the two ribosomal subunits and plays a critical role in assessment of correct base-pairing between the codon and anticodon and the subsequent structural rearrangement that occurs when a correct pair is recognized. The high sequence conservation of EF-Tu and S12 can be attributed to both the need to interact with multiple binding partners and to the intricacy of their functions, which require exquisite sensitivity to the shape of the codon-anticodon pair [15], and apparently place strong constraints on numerous areas of the protein, thus preventing drift due to accumulation of mutations. Strikingly, neither DNA polymerases nor RNA polymerases in this proteome show such stringent conservation, even though high fidelity is also important in replication and transcription.

The CodaChrome heat map shows that the chaperones DnaK [16], GroEL [17] and the ClpXP protease are also particularly conserved (Figure 2b and c). Although these proteins are unrelated, they all interact with a number of “client” proteins, undergo multiple conformational changes, and couple the hydrolysis of ATP to a downstream process that maintains the integrity of proteins in the cytoplasm. For example, ClpXP proteases [18], which are found widely in bacteria, as well as in mitochondria and chloroplasts, consist of six ClpX monomers in a ring stacked upon two heptameric rings of ClpP. The ATPase subunit of ClpX recognizes degradation tags in proteins (such as the ssrA tag that is added to incomplete proteins released from stalled ribosomes), unfolds the protein and translocates it to the cavity in the rings of ClpP protease. ClpX recognizes over 100 cellular proteins [19,20]. Mutations in ClpX increase or decrease activity toward certain substrates. Thus it appears that its structure is an evolutionary compromise that allows it to function with a large number of substrates at a reasonable level, even though not optimally with any one [18]. The important functions and complex structures of these multimeric proteins, along with the requirement for interaction with numerous client proteins, have evidently severely constrained the divergence of sequence over vast periods of time.

### Identification of Fast-Clock Genes using CodaChrome

Sequencing of 16S rRNA from environmental or clinical samples allows identification of microbes down to the genus and often species level, but often cannot distinguish between strains and closely related species [21-23]. It is clear that understanding the roles of particular microbes in ecosystems requires finer granularity for classification. Addressing this challenge requires identification of a locus that is moderately conserved across a genus or species of interest but that changes more quickly than the sequence of the 16S ribosomal subunit.

**Figure 3:**
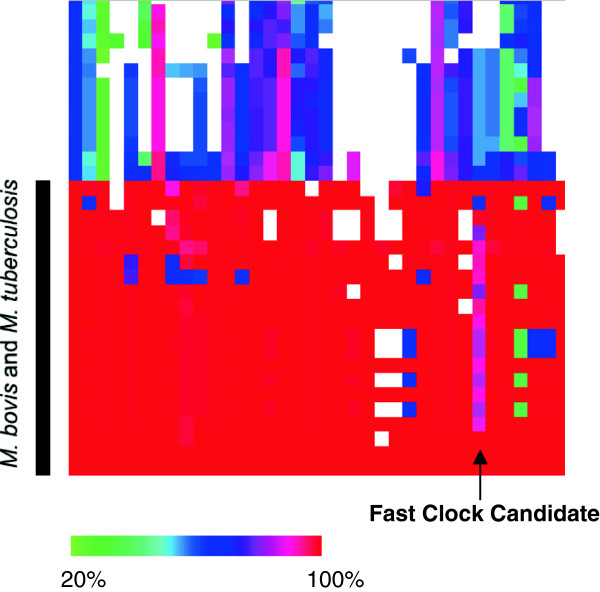
**Use of CodaChrome to identify fast-clock genes.** Enlargement of a region of a CodaChrome heat map with *Mycobacterium tuberculosis* H37Rv loaded as the seed sequence and with the PID threshold set to 20%. The rows were sorted by overall percent identity.

CodaChrome is an ideal tool for identification of “fast-clock” genes within a lineage of interest. The user simply loads the species of interest as the seed, sorts by whole proteome identity, and then looks for pillars of proteins that show marked variation in color. For example, we identified a fast-clock gene useful for the subtyping of several strains of *Mycobacterium tuberculosis* and *Mycobacterium bovis,* all of which possess identical 16S rRNA sequences*. Mycobacterium tuberculosis* H37Rv was loaded as the seed organism, the percent identity threshold was set to 20% and rows were sorted by whole-proteome percent identity. On the bottom of the heat map in Figure 3, 19 red rows represent the proteins of 19 strains of *Mycobacterium* that are closely related to the seed organism*.* An ideal fast-clock gene will show significant variation throughout the 19 strains; one such gene, which encodes a PPE family protein, is easily identified by the color variation in the column (see Figure 3). A multiple sequence alignment of these sequences confirms the existence of many subtype-specific SNPs and indels. Additional file 1: Table S1 contains a matrix of pairwise counts of identical positions, including gaps, in the multiple sequence alignment. Fast-clock genes could be used for rapid identification of strains in clinical or environmental samples based upon differential PCR amplification of variable regions.

The identification of fast-clock genes by visual inspection of the CodaChrome heat map begs the question of *why* such genes are under selection for unusual variability. Examination of this question can reveal evolutionarily important selective pressures exerted by the environment(s) in which the microbe lives. For example, PPE family proteins in Mycobacteria have been implicated in antigenic variation [24].

### Identification of the evolutionary history of an indel using CodaChrome

**Figure 4:**
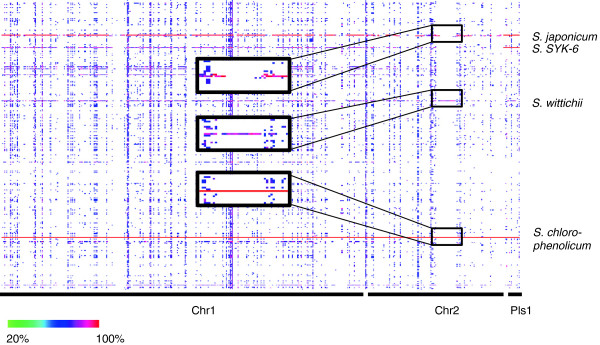
**Use of CodaChrome to investigate the evolutionary history of an indel.** A CodaChrome heat map is shown in which the proteome of *Sphingobium chlorophenolicum* was loaded as the seed sequence and the rows were not sorted. The boxed regions show a section of the proteome of *S. chlorophenolicum* that lacks orthologs in the closely related *S. japonicum* but has orthologs in the more distantly related *S. wittichii*.

Insertions and deletions are common genetic changes that can occur on the scale of single genes or on the scale of hundreds of genes. They are enormously important in determining characteristics such as virulence, metal tolerance, nutrient requirements, and degradative capabilities. Insertions and deletions are often combined into a single category termed “indels” because in pairwise alignments it is impossible to distinguish whether the observed difference is the result of a deletion from one sequence or an insertion into the other. Additional information from the sequences of more distantly related species is required to resolve the cause of the difference. If the sequence of interest is absent from a more distant relative, parsimony suggests that the indel was absent from the most recent common ancestor as well and arose by insertion in one lineage. Conversely, if the sequence is present in the distant relative, it is more likely that the indel was present in the most recent common ancestor as well and that the indel arose by deletion in the other lineage. The strength of this conclusion can be augmented if there are numerous relatives for comparison. CodaChrome facilitates such analyses by generating a single heat map sufficient for both identification and analysis of indels. Figure 4 shows a CodaChrome heat map in which *Sphingobium chlorophenolicum* L-1 was loaded as the seed organism with the percent identity threshold set to 70%. The rows are not sorted, and so remain ordered alphabetically by their GenBank accession numbers. *Sphingobium japonicum* is the most closely related organism included in the database as judged by the red row indicating very high sequence identity extending across nearly all of the heat map. While the majority of proteins in *S. chlorophenolicum* share over 90% identity with homologs in *S. japonicum*[8], close comparison reveals several large discrepancies, such as the one in the boxed regions of Figure 4. These regions could correspond to a large insertion in the genome of *S. chlorophenolicum* or a large deletion from the genome of *S. japonicum.* The proteome-wide view provided by CodaChrome resolves this question by illustrating that proteins in the boxed segment of the *S. chlorophenolicum* proteome are highly similar to those found in another organism in the database, *Sphingomonas wittichii. S. wittichii* is a more distant relative of *S. chlorophenolicum* and *S. japonicum.* Since the proteins included in the boxed region are present in *S. wittichii,* the data support the hypothesis that this segment was present in the common ancestor of *S. wittichii, S. japonicum* and *S. chlorophenolicum* and was lost in the lineage leading to *S. japonicum.*

Examination of questions regarding evolutionary history such as this one are facilitated by CodaChrome because the proteomes encoded by all finished bacterial genomes are simultaneously visualized. Thus, there is no requirement to select, *a priori*, a subset of genomes that is suspected of harboring informative relationships, as is the case when utilizing whole genome alignment software to approach this problem.

### CodaChrome facilitates analysis of the pan-genome of bacterial species

**Figure 5:**
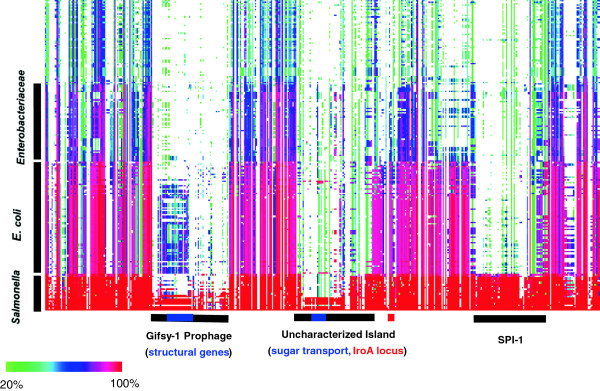
**Use of CodaChrome to identify genomic islands in closely related organisms.** The figure shows an expansion of a section of the CodaChrome heat map shown in Figure 1.

CodaChrome is a powerful tool for tracking the movements of blocks of genes that play roles in processes such as virulence, biodegradation, and metabolism. Figure 5 shows a region of the CodaChrome heat map in which the proteome of *Salmonella enterica* 14028S was loaded as the seed organism and the rows were sorted by average percent identity shared with *S. enterica* 14028S with the percent identity threshold set at 20%. The proteomes of the 25 species of *Salmonella* for which finished genome sequences are available sorted to the bottom of the image as a cluster of almost entirely red rows. Above this bright red cluster is a cluster of red and purple rows that represent the proteomes of various strains of *E. coli*. The rows of *E. coli* proteomes are striated with vertical white gaps that represent sections of the *Salmonella enterica* 14028S genome that encode proteins with no clear homology to any *E. coli* proteins. Above the *E. coli* cluster is a cluster of more distantly related Enterobacteriaceae. When sorted this way, large segments that are present in some but not all closely related strains of *Salmonella* are apparent (see horizontal bars).

One of the striking features in the heat map corresponds to the lambdoid prophage Gifsy-1. This prophage is present in about half of the strains of Salmonellae and is largely missing from the *E. coli* cluster aside from a few moderately conserved homologs of the phage structural proteins. It is entirely missing from the more distantly related cluster of *Enterobacteriaceae* strains. Phage are abundant in nature, and in aquatic habitats they typically out-number microbes by a ratio of 10:1 [25-27]. Most microbial genomes carry relics of past phage infections in the form of integrated prophages. As clearly revealed in CodaChrome heat maps, these prophages are often the most unique segments of a genome. Under some situations, this characteristic makes them useful loci for strain identification [28,29]. Additionally, some prophages, such as Gifsy-1 in *S. enterica,* have been implicated in pathogenicity [30]. CodaChrome provides a convenient way to visualize the host range of the phage precursor. Simply selecting the cluster of phage proteins and clicking “sort” will reorder the rows such that all replicons that contain similar proteins will sort to the bottom of the heat map.

Prophage insertions are not the only insertions with clinical relevance to leave a clear signature in CodaChrome. Pathogenicity islands also leave a unique pattern. Pathogenicity islands are segments of horizontally transferred genetic material that encode proteins involved in virulence. Pathogenicity islands have been identified in many pathogens, including species of *Escherichia, Vibrio, Listeria, Shigella, Yersina, Salmonella, Pseudomonas, Erwinia, Helicobacter,* and *Agrobacterium*[31]. These islands encode proteins involved in a range of processes, including adherence, toxin catabolism, iron uptake, and secretion. Pathogenicity islands range in size from a handful of genes to hundreds. They often have anomalous GC content or codon usage, both hallmarks of horizontal gene transfer. However, when the donor and recipient of the genetic material share similar GC content and codon usage, or when the recipient has had sufficient time to equilibrate these properties, these markers may become more difficult to detect or disappear entirely [32]. The signature of abnormal inheritance that is visualized in CodaChrome remains after these other markers of horizontal gene transfer have been ameliorated. An example of the signature caused by the *Salmonella* Pathogenicity Island 1 (SPI-1) is labeled with a black bar in Figure 5. SPI-1 encodes 47 proteins, most of which are involved in assembly or function of the Type III secretion system [33], or are effector proteins that are injected into host cells through the needle of the secretion complex.

Like the prophage on the left of Figure 5, SPI-1 is conspicuously lacking from the closely related cluster of *E. coli* as well as the cluster of enterobacteriaceae. Unlike the prophage, however, SPI-I is present in all sequenced strains of *Salmonella*, reflecting its essential role in *S. enterica* pathogenesis. Figure 5 also shows a third, largely uncharacterized region of anomalous sequence conservation. This region encompasses an odd collection of genes, including genes involved in iron metabolism, sugar transport and many conserved proteins of unknown function.

### Use of CodaChrome as a discovery tool

**Figure 6:**
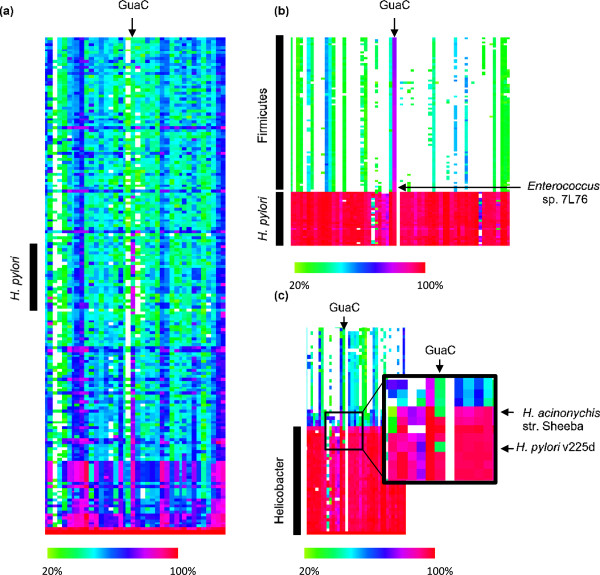
**CodaChrome heat maps reveal unexpected sequence relationships. a)** A region of the CodaChrome heat map in which *B. subtilus* was loaded as the seed organism, the rows were sorted according to whole proteome identity, and alignments with pairwise percent identities below 20% were filtered out. **b)** A region of the CodaChrome heat map in which *H. pylori* P12 was loaded as the seed organism, the rows were sorted according by identity to GuaC of *H. pylori* P12, and alignments with pairwise percent identities below 20% were filtered out. **c)** Enlargement of the region around GuaC of a CodaChrome heat map with *H. pylori* P12 loaded as the seed proteome and rows sorted by whole proteome percent identity.

The previous examples have illustrated the use of CodaChrome for analysis of biologically interesting questions. However, CodaChrome can also be used as a discovery tool due to its ability to reveal patterns in sequence conservation that are easily recognized by the human eye. These striking patterns indicate relationships, or in some cases, the absence of expected relationships, that might not have been previously recognized. An example is shown in Figure 6a. When the proteome of the Firmicute *Bacillus subtilus* was used as the seed in CodaChrome and replicons were sorted by average percent identity to the entire seed proteome with a percent identity cutoff of 20%, we noticed a vertical stripe of red and pink standing out in a region far above the horizontal band of red and pink corresponding to proteomes closely related to that of *B. subtilis* (Figure 6a). This vertical stripe is due to unexpectedly high sequence identity between *B. subtilis* guanosine reductase (GuaC) and homologous proteins in several strains of the Proteobacterium *Helicobacter pylori.*

Guanosine reductase is involved in purine salvage. It converts guanosine monophosphate to inosine monophosphate (IMP), which in turn can be converted to adenosine monophosphate. Since *H. pylori* lacks the pathway for *de novo* synthesis of inosine monophosphate, its ability to salvage purines from its environment is essential for growth [34]. GuaC from *B. subtilis* is 81% identical to GuaC from *H. pylori*; this is more than twice the level of percent identity shared by most homologous proteins in these bacteria as assessed by the color of the rows corresponding to *H. pylori* in the CodaChrome graph in which *B. subtilis* is the seed organism. Not even highly conserved proteins such as S12 and EF-Tu share such a high percent identity; S12 and EF-Tu in the two bacteria are 68% and 74% identical, respectively.

We investigated this surprising relationship further by loading the proteome of *H. pylori* as the seed sequence (Figure 6b) and sorting the heat map by percent identity to *H. pylori* GuaC has with a PID cutoff of 20%. This view shows that *H. pylori* GuaC has closely related homologs in many Firmicutes; the closest homolog is found in *Enterococcus sp.* 7 L76*.* Additional file 1: Figure S2 shows values for the percent identity between GuaC from *Enterococcus sp.* 7 L76 and its closest homolog in other microbes represented on a bacterial phylogenetic tree [35]. Indeed, *Enterococcus sp.* 7 L76 GuaC is more closely related to homologs in *H. pylori* than to homologs in many closely related species of Firmicutes.

From this pattern in CodaChrome, we infer that the gene encoding GuaC was transferred from a recent ancestor of *Enterococcus sp.* 7 L76 to a recent ancestor of *H. pylori. H. pylori* colonizes the stomach in humans, while many species of *Enterococcus* are found lower in the intestinal tract [36]. *H. pylori* might have acquired a gene encoding GuaC from lysed enterococci that had been ingested through food or fecal-oral contamination and killed by the highly acidic environment of the stomach. The acquisition of GuaC, as well as other enzymes involved in purine salvage, may have allowed this bacterium to lose the genes required for *de novo* purine synthesis as it adapted for growth within the nutrient-rich environment of the stomach.

Re-sorting the CodaChrome heat map by percent identity across the entire proteome reveals another surprising finding (Figure 6c). The closest homologs to *H. pylori* GuaC are unexpectedly distant in two closely related species of *Helicobacter*; *H. acinonychis* str. Sheeba, a gastric pathogen of big cats that is believed to have jumped from humans to large felines about 200,000 years ago [37], and *H. pylori* v225d, a clinical strain isolated from a Piaroa Amerindian with acute gastritis [38]. The proteins corresponding to the green blocks indicated by arrows in Figure 6c are annotated as IMP dehydrogenases. These annotations are undoubtedly correct, as these proteins are >97% identical to IMP dehydrogenases from other strains of *H. pylori.* Thus, the mechanisms by which these bacteria salvage purines for synthesis of nucleotides are unknown.

## Conclusion

CodaChrome is powerful enough to visualize relationships between thousands of bacterial genomes, yet sensitive enough to visualize the deletion of a single protein from a single species. CodaChrome builds users’ intuition about the relationships between genomes. It establishes what is “normal” and in doing so provides a baseline for identifying what is unusual about proteins within a particular genome. Unlike many visualization tools in which data are used to render a static image, CodaChrome is interactive. Patterns are not just visualized, but can easily be investigated in more detail by sorting the rows in various ways, zooming in on a region of interest, and/or mousing over individual blocks to obtain specific information about the pair-wise relationship represented by the block.

Sorting the CodaChrome heat map by average percent identity across the entire proteome provides users with an empirical estimate of the phylogenetic distances between the proteome of the seed organism and the proteomes of all other organisms in the database. Sorting by subsets of the seed proteome allows users to investigate individual proteins or clusters of proteins that have experienced atypical selection pressures or horizontal gene transfer.

As demonstrated in the examples above, this tool allows investigation of a broad range of biological questions at scales ranging from individual proteins to groups of proteins transferred *en bloc* to the entire proteome. Further, CodaChrome visualizations contain more information than the individual alignments of which they are composed. Visualizing all of the alignments simultaneously forms patterns indicative of biological phenomena that could not easily be identified by examining the alignments individually.

## Availability and requirements

Documentation, source code, pre-compiled binaries for common operating systems, pre-computed CodaChrome matrix files for many bacterial genomes of interest including those described in this paper and a tutorial for the use of CodaChrome are available at http://www.sourceforge.com/p/codachrome. CodaChrome is platform-independent and programmed in C++/QT 5.0. It is licensed under the GNU GPL version 3.

## Competing interest

The authors declare that they have no competing financial interests.

## Authors’ contributions

JR, DK, RD, and SC conceived the project and designed the experiments and software. JR wrote the source code. JR and SC wrote the manuscript. All authors read and approved the final manuscript.

## Supplementary Material

Additional file 1: Figure S1 Schematic of the method of proteome comparison used by CodaChrome. **Table S1.** Pairwise percent identities between homologs of PPE34 (YP_177655.1) in closely related strains of Mycobacteria. **Figure S2.** Mapping of the pair-wise sequence identities between GuaC from *Enterococcus faecalis* and the closest homolog in other representative bacteria onto a phylogenetic tree. Click here for file
