# Supplementary material for: CodaChrome: a tool for the visualization of proteome conservation across all fully sequenced bacterial genomes
Source: BMC Genomics. 2014 Jan 24;15:65. doi: 10.1186/1471-2164-15-65 (PMC3908345; doi:10.1186/1471-2164-15-65)
Supplement: Additional file 1: Figure S1 — Schematic of the method of proteome comparison used by CodaChrome. Table S1. Pairwise percent identities between homologs of PPE34 (YP_177655.1) in closely related strains of Mycobacteria. Figure S2. Mapping of the pair-wise sequence identities between GuaC from Enterococcus faecalis and the closest homolog in other representative bacteria onto a phylogenetic tree. [file 1471-2164-15-65-S1.docx]

**CodaChrome: a tool for the visualization of proteome conservation across all fully sequenced bacterial genomes**

Joe Rokicki^1^, David Knox^1,3^, Robin D. Dowell^1,2,*^, Shelley D. Copley^1,*^

**Additional file 1: Figure S1.** Schematic of the method of proteome comparison used by CodaChrome.

**Additional file 1: Table S1.** Pairwise percent identities between homologs of PPE34 (YP_177655.1) in closely related strains of Mycobacteria.

**Additional file 1: Figure S2.** Mapping of the pair-wise sequence identities between GuaC from *Enterococcus faecalis* and the closest homolog in other representative bacteria onto a phylogenetic tree.

**
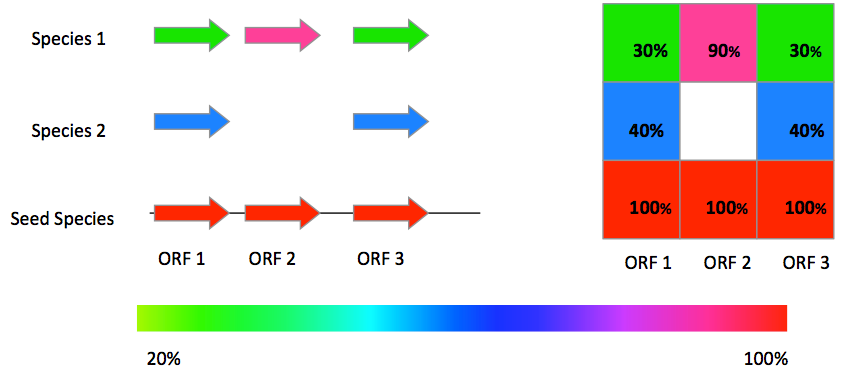
**

Figure S1. Schematic of the proteome visualization scheme used by CodaChrome. Arrows on the left of the figure represent ORFs. The boxes on the right represent the pairwise identities between the two proteins being compared. Note that the proteins in the seed species are shown in the order in which the genes occur in the genome, but the proteins in species 1 and 2 are not.

**Table S1.** Pair-wise percent identities between homologs of PPE34 (YP_177655.1) in closely related strains of Mycobacteria. *

| **Percent Identities** | F11 | GM041182 | AF2122/97 | Mexico | Tokyo-172 | Pasteur-1173P2 | CTRI-2 | CDC1551 | UT205 | KZN1435 | KZN4207 | KZN605 | CCDC5079 | H37Rv | H37Ra | RGTB423 | CCDC5180 |
| --- | --- | --- | --- | --- | --- | --- | --- | --- | --- | --- | --- | --- | --- | --- | --- | --- | --- |
| F11 | 100 | 81 | 77 | 78 | 78 | 78 | 92 | 96 | 96 | 96 | 96 | 96 | 50 | 95 | 95 | 95 | 87 |
| GM041182 | 81 | 100 | 96 | 92 | 92 | 92 | 77 | 81 | 79 | 77 | 77 | 77 | 31 | 78 | 78 | 78 | 90 |
| AF2122/97 | 77 | 96 | 100 | 91 | 91 | 91 | 73 | 77 | 75 | 73 | 73 | 73 | 28 | 74 | 74 | 74 | 86 |
| Mexico | 78 | 92 | 91 | 100 | 100 | 100 | 77 | 81 | 79 | 76 | 76 | 76 | 31 | 76 | 76 | 76 | 87 |
| Tokyo-172 | 78 | 92 | 91 | 100 | 100 | 100 | 77 | 81 | 79 | 76 | 76 | 76 | 31 | 76 | 76 | 76 | 87 |
| Pasteur-1173P2 | 78 | 92 | 91 | 100 | 100 | 100 | 77 | 81 | 79 | 76 | 76 | 76 | 31 | 76 | 76 | 76 | 87 |
| CTRI-2 | 92 | 77 | 73 | 77 | 77 | 77 | 100 | 92 | 94 | 94 | 94 | 94 | 52 | 89 | 89 | 89 | 84 |
| CDC1551 | 96 | 81 | 77 | 81 | 81 | 81 | 92 | 100 | 98 | 95 | 95 | 95 | 49 | 91 | 91 | 91 | 87 |
| UT205 | 96 | 79 | 75 | 79 | 79 | 79 | 94 | 98 | 100 | 98 | 98 | 98 | 52 | 93 | 93 | 93 | 87 |
| KZN1435 | 96 | 77 | 73 | 76 | 76 | 76 | 94 | 95 | 98 | 100 | 100 | 100 | 54 | 93 | 93 | 93 | 85 |
| KZN4207 | 96 | 77 | 73 | 76 | 76 | 76 | 94 | 95 | 98 | 100 | 100 | 100 | 54 | 93 | 93 | 93 | 85 |
| KZN605 | 96 | 77 | 73 | 76 | 76 | 76 | 94 | 95 | 98 | 100 | 100 | 100 | 54 | 93 | 93 | 93 | 85 |
| CCDC5079 | 50 | 31 | 28 | 31 | 31 | 31 | 52 | 49 | 52 | 54 | 54 | 54 | 100 | 50 | 50 | 50 | 39 |
| H37Rv | 95 | 78 | 74 | 76 | 76 | 76 | 89 | 91 | 93 | 93 | 93 | 93 | 50 | 100 | 100 | 100 | 88 |
| H37Ra | 95 | 78 | 74 | 76 | 76 | 76 | 89 | 91 | 93 | 93 | 93 | 93 | 50 | 100 | 100 | 100 | 88 |
| RGTB423 | 95 | 78 | 74 | 76 | 76 | 76 | 89 | 91 | 93 | 93 | 93 | 93 | 50 | 100 | 100 | 100 | 88 |
| CCDC5180 | 87 | 90 | 86 | 87 | 87 | 87 | 84 | 87 | 87 | 85 | 85 | 85 | 39 | 88 | 88 | 88 | 100 |

* Red, *M. tuberculosis* strains; green, *M. africanum* strains; blue, *M. bovis* BCG strains.

**Figure S2**. Mapping of the pair-wise sequence identities between GuaC from *Enterococcus faecalis* and the closest homolog in other representative bacteria onto a phylogenetic tree.
